# Macrolide Resistance of *Mycoplasma pneumoniae,* South Korea, 2000–2011

**DOI:** 10.3201/eid1908.121455

**Published:** 2013-08

**Authors:** Ki Bae Hong, Eun Hwa Choi, Hoan Jong Lee, Seong Yeon Lee, Eun Young Cho, Jae Hong Choi, Hyun Mi Kang, Jina Lee, Young Min Ahn, Yeon-Ho Kang, Joon-Ho Lee

**Affiliations:** Seoul National University Hospital, Seoul, South Korea (K.B. Hong, E.H. Choi, H.J. Lee, S.Y. Lee, E.Y. Cho, J.H. Choi, H.M. Kang);; Seoul National University College of Medicine, Seoul (E.H. Choi, H.J. Lee);; Seoul National University Bundang Hospital, Seongnam, South Korea (J. Lee);; Seoul Eulji Hospital, Seoul (Y.M. Ahn);; Korea Centers for Disease Control and Prevention, Seoul (Y.-H. Kang);; Kangwon National University Hospital, Chuncheon, South Korea (J.-H. Lee)

**Keywords:** *Mycoplasma pneumoniae*, pneumonia, resistance, macrolide, respiratory infections, South Korea, tuberculosis and other mycobacteria

## Abstract

In Korea, *Mycoplasma pneumoniae* was detected in 255/2,089 respiratory specimens collected during 2000–2011; 80 isolates carried 23S rRNA gene mutations, and 69/123 culture-positive samples with the mutation were resistant to 5 macrolides. During 2000–2011, prevalence of the mutation increased substantially. These findings have critical implications for the treatment of children with mycoplasma pneumonia.

*Mycoplasma pneumoniae* is 1 of the most common causes of community-acquired pneumonia in children and young adults (*1*). Epidemics of mycoplasma pneumonia typically occur every 4–7 years; however, epidemics have occurred every 3–4 years in South Korea (*2*,*3*). The first-line treatment for mycoplasma pneumonia is macrolide antimicrobial drugs, but macrolide-resistant infections have been recognized in conjunction with an increase in cases in children in Japan, China, Germany, France, Israel, and the United States (*1*,*4*–*10*). Because of the risk to children administered tetracycline and fluoroquinolone (nonmacrolide drugs), *M. pneumoniae* resistance to macrolide drugs has critical implications for the treatment of mycoplasma pneumonia in children. This study was conducted to identify the prevalence of macrolide resistance among *M. pneumoniae* strains isolated from children with lower respiratory tract infections (LRTIs) during 4 consecutive epidemics (2000–2011) in South Korea.

## The Study

A total of 2,089 respiratory samples were tested for the presence of *M. pneumoniae*. Of these, a total of 378 were archived samples collected during epidemics in 2000 (71 samples), 2003 (112 samples), and 2006 (195 samples), and 1,711 were samples collected and tested during August 2010–December 2011. Specimens from the 2010–2011 epidemic were collected from children at Seoul National University Children’s Hospital, Seoul National University Bundang Hospital, and Seoul Eulji Hospital. All samples were obtained from children (median age 5 years, range 6 months–18 years) with a diagnosis of community-acquired LRTI.

P1 adhesin was amplified by PCR for the detection of *M. pneumoniae* from the 378 archived samples. *M. pneumoniae* was cultivated by using pleuropneumonia-like organism broth and agar for the 1,711 samples collected during 2010–2011. Media were incubated aerobically at 37°C for 6 weeks. Plates were observed daily to identify change in the color of the broth from red to transparent orange. When the color changed, the samples were subcultured on agar plates. Spherical *M. pneumoniae* colonies were observed by using a microscope.

For the cultured *M. pneumoniae* isolates, we amplified domain V of the 23S rRNA gene by PCR; for the archived samples, we extracted DNA. For PCR, we used primers MP23SV-F 5′-TAACTATAACGGTCCTAAGG-3′ and MP23SV-R 5′-ACACTTAGATGCTTTCAGCG-3′. The PCR products were sequenced to identify mutations. Sixty-four of the *M. pneumoniae*–positive samples from 2000 and 2003 had been previously tested for mutations in the 23S rRNA gene.

Minimum inhibitory concentrations (MICs) were measured by using the microdilution method in triplicate for the following antimicrobial agents: erythromycin, clarithromycin, azithromycin, roxithromycin, josamycin, tetracycline, doxycycline, levofloxacin, moxifloxacin, and ciprofloxacin. MIC was defined as the lowest antimicrobial drug concentration at which the media color did not change at the time when the color of the positive control media (containing *M. pneumoniae* strains only) changed (*11*).

*M. pneumoniae* was detected in 255 (12.2%) of 2,089 clinical samples; 132 (51.8%) of the positive samples were among the 378 archived samples, and 123 (48.2%) were among the 1,711 samples from 2010–2011. For the 132 archived samples, *M. pneumoniae* was detected by PCR: 30 (22.8%) were among the 71 samples from 2000, 34 (25.8% were among the 112 samples from 2003, and 68 (51.5%) were among the 195 samples from 2006. For the 123 samples from 2010–2011, *M. pneumoniae* was detected by culture.

Overall, 80 (31.4%) of the 255 *M. pneumoniae*–positive samples carried mutations in the 23S rRNA gene. Of these, 78 had the A2063G transition and 2 exhibited the A2064G transition. The prevalence of the 23S rRNA mutation increased significantly over the 4 consecutive epidemics, as follows: 2000 epidemic, 0 of 30 samples; 2003 epidemic, 1 (2.9%) of 34 samples; 2006 epidemic, 10 (14.7%) of 68 samples; and 2010–2011 epidemic, 25 (47.2%) of 53 samples in 2010 and 44 (62.9%) of 70 samples in 2011 (p<0.001 for trend) (Figure).

**Figure:**
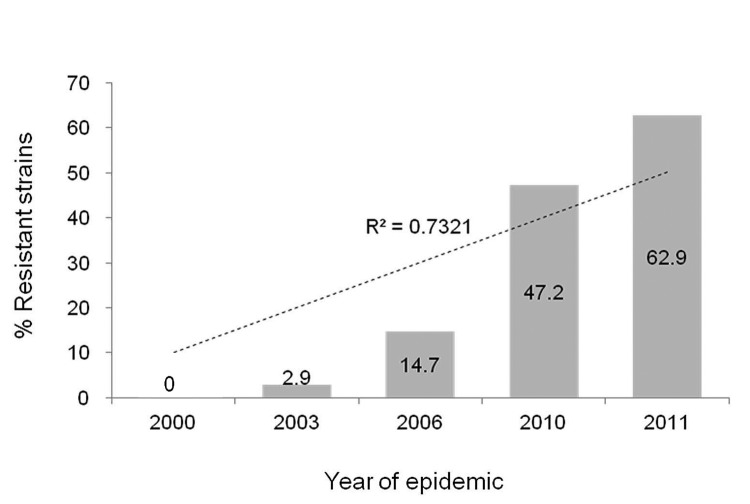
Increased prevalence of macrolide resistance of *Mycoplasma pneumoniae* strains isolated from children during epidemics of lower respiratory tract infections, South Korea, 2000–2011. During the 2000 epidemic, 0 of 30 strains were resistant, but during the epidemics of 2003 and 2006, 1 of 34 and 10 of 68 strains, respectively, showed resistance. During the 2010–2011 outbreak, 25 of 53 (2010) and 44 of 70 (2011) strains were resistant. Numbers on the bars are the percentages of resistant strains for each year.

Among 123 samples culture-positive for *M. pneumoniae*, 69 that carried the 23S rRNA mutation exhibited significantly higher MIC_50_ (MIC for 50% of strains) and MIC_90_ when tested with 5 macrolides, compared with 54 strains that lacked the mutation. For example, the MIC_50_ and MIC_90_ of erythromycin were 16 µg/mL and 128 µg/mL, respectively, for strains with the 23S rRNA mutation and 0.001 µg/mL and 0.002 µg/mL, respectively, for strains without the mutation (p<0.0001) (Table 1). All 123 *M. pneumoniae* strains were susceptible to nonmacrolide antimicrobial drugs, including tetracycline, doxycycline, levofloxacin, ciprofloxacin, and moxifloxacin (Table 2).

**Table 1:** MICs of macrolide antimicrobial drugs for 123 *Mycoplasma pneumoniae* strains in a study of macrolide resistance, South Korea, 2000–2011*

Macrolides	Strains with 23S rRNA mutation, n = 69				Strains without 23S rRNA mutation, n = 54		
	Range	MIC_50_	MIC_90_		Range	MIC_50_	MIC_90_
Erythromycin	2 to >128	16	128		0.001 to 0.004	0.001	0.002
Clarithromycin	8 to >128	64	128		0.001 to 0.002	0.001	0.002
Roxithromycin	0.008 to 128	8	32		0.001 to 0.008	0.001	0.004
Azithromycin	1 to 64	8	16		0.001 to 0.001	0.001	0.001
Josamycin	1 to 8	4	8		0.001 to 0.016	0.001	0.008

*MIC_50_ and MIC_90_ are minimum inhibitory concentrations at which 50% and 90% of the isolates, respectively, were inhibited by the drug. In each instance, p<0.0001.

**Table 2:** MICs of tetracyclines and fluoroquinolones for *Mycoplasma pneumoniae* strains in a study of macrolide resistance, South Korea, 2000–2011*

Antimicrobial drug	Strains with 23S rRNA mutation, n = 69				Strains without 23S rRNA mutation, n = 54		
	Range	MIC_50_	MIC_90_		Range	MIC_50_	MIC_90_
Tetracyclines							
Tetracycline	0.016 to 0.5	0.06	0.25		0.016 to 0.5	0.06	0.25
Doxycycline	0.002 to 0.125	0.06	0.06		0.004 to 0.125	0.03	0.06
Fluoroquinolones							
Levofloxacin	0.016 to 0.5	0.25	0.25		0.016 to 0.5	0.25	0.5
Ciprofloxacin	0.125 to 1.0	0.5	1.0		0.06 to 1.0	0.5	1.0
Moxifloxacin	0.008 to 0.06	0.016	0.06		0.004 to 0.06	0.016	0.06

*MIC_50_ and MIC_90_ are minimum inhibitory concentrations at which 50% and 90% of the isolates, respectively, were inhibited by the drug.

Macrolide resistance is associated with point mutations in domain V of the *M. pneumoniae* 23S rRNA gene, especially those corresponding to A2063G or A2064G transitions (*4*,*5*,*7*). Thus, emergence of macrolide-resistant strains may result in treatment failure of *M. pneumoniae* infections (*4*).

We did not detect macrolide resistance among *M. pneumoniae* strains collected during 2000; thereafter, the prevalence of macrolide resistance remained low through the 2003 epidemic. Macrolide resistance then increased to 14.7% during the epidemic of 2006 and to 56.1% during the epidemic of 2010–2011, as indicated by substantially higher MICs against macrolide agents in association with the presence of the 23S rRNA gene mutation in *M. pneumoniae* isolates.

Macrolide resistance has been detected with increasing frequency in many parts of the world, highlighting the importance of knowing the geographic distribution and temporal patterns of macrolide-resistant *M. pneumoniae*. After the first isolation of a macrolide-resistant strain in 2001, Japan reported a dramatic increase in macrolide resistance among children with mycoplasma pneumonia, and in 2011 resistance was >80% (*12*,*13*). China identified an 83%–92% prevalence of macrolide-resistant *M. pneumoniae* isolates (*6*,*9*). In contrast, France identified only 2 resistant *M. pneumoniae* isolates during 1994–2006, and the United States reported a 30% prevalence of macrolide-resistant strains (*10*). Israel reported that in 2010, ≈30% of *M. pneumoniae* isolates carried an A2063G transition in domain V of the 23S rRNA gene (*8*). In Italy, 26% of *M. pneumoniae*–infected children harbored strains with point mutations in domain V of the 23S rRNA gene (*14*). Thus, there is great variability in the prevalence of macrolide resistance in *M. pneumoniae* isolates.

## Conclusions

A key finding of this study is the increasing prevalence of macrolide resistance over time. Several factors may have led to this increase. First, the increased use of macrolide antimicrobial drugs may be responsible for the development and spread of macrolide resistance. A recent study showed a correlation between increased use of oral macrolides and an increase in macrolide resistance by selective pressure in *M. pneumoniae* and other respiratory pathogens (*13*). A comprehensive trend analysis of the national data showed an increase in macrolide use in the community (expressed in defined daily doses [DDD]/1,000 inhabitants/day) during 2005–2009 (*15*). Penicillins and cephalosporins are the 2 most frequently used classes of oral antimicrobial drugs. There were decreasing trends in penicillin use and a subtle increase in cephalosporin use during 2005–2009. Macrolide use remained steady until 2007; however, there was an increase of >30% in DDD/1,000 inhabitants/day between 2007 (2.5 DDD/1,000 inhabitants/day) and 2009 (3.3 DDD/1,000 inhabitants/day). Our data on macrolide use do not fully explain the 10-year change in *M. pneumoniae* resistance to macrolide drugs because data were available only for 2005–2009. In addition, the spread of resistant strains could have been facilitated by other factors, such as high population density or geographic closeness with the 2 neighboring countries, where resistant strains were highly prevalent.

We found an increasing prevalence of the 23S rRNA gene mutation in *M. pneumoniae* isolates during 2000–2011 in South Korea. We did not address the clinical issues regarding antimicrobial drug choices for macrolide-resistant mycoplasma pneumonia or compare the clinical outcomes for macrolide-resistant and macrolide-sensitive infections; nevertheless, we believe that the evidence of a recent increase in macrolide resistance provides guidance for additional clinical investigations and new therapeutic strategies. The incidences of macrolide-resistant *M. pneumoniae* infection should be carefully monitored, particularly among children, for whom treatment can be challenging. Further studies are needed to evaluate the clinical significance of macrolide-resistant *M. pneumoniae* pneumonia.
